# Radiological and functional outcomes of Reverdin Isham osteotomy in moderate Hallux Valgus: a systematic review and meta-analysis

**DOI:** 10.1038/s41598-024-65440-3

**Published:** 2024-06-26

**Authors:** Victoria Sanchís-Soria, Elena Nieto-González, Eduardo Nieto-García, Nadia Fernández-Ehrling, Javier Ferrer-Torregrosa, Rubén Lorca-Gutiérrez

**Affiliations:** 1 Doctorate School, Catholic University of Valencia San Vicente Mártir, 46001 Valencia, Spain; 2 Podiatry Department, Faculty of Medicine and Health Sciences, Valencia Catholic University San Vicente Mártir, Valencia, Spain

**Keywords:** Minimally invasive surgical procedures, Reverdin-Isam, Osteotomy, Hallux Valgus, Outcomes research, Trauma

## Abstract

This systematic review and meta-analysis addresses the effects of minimally invasive surgical techniques, specifically the Reverdin Isham osteotomy, on functional and radiological outcomes in patients with moderate Hallux Valgus, a common foot deformity. The review included randomized and non-randomized controlled trials, as well as case reports, assessing the osteotomy in adults with moderate to severe Hallux Valgus. Searches were conducted in electronic databases such as MEDLINE and Web of Science up until July 2023, and the Joanna Briggs Institute’s critical appraisal tool was used to assess the risk of bias. Meta-analytical analyses employed a random-effects model with small-sample correction, presenting results as standardized mean differences and mean differences with 95% confidence intervals. Seven studies involving 554 patients and 643 operated feet were included, showing significant improvements in AOFAS scores (an average improvement of 36 points from 28.61 to 45.16) and reductions in radiological angles such as the distal metatarsal angle and hallux valgus angle post-surgery (IMA improved by − 3.07° from − 4.68 to − 1.46, DMAA by − 6.12° from − 9.52 to − 2.71, and HVangle by − 15.27° from − 17.98 to − 12.57). Despite these positive outcomes, most studies exhibited risks of bias and other methodological limitations, impacting the generalizability of the results. Overall, the findings highlight the efficacy of the Reverdin Isham osteotomy in improving both functional and radiological parameters in patients with moderate Hallux Valgus, although further research is warranted to solidify these results. No specific funding was received for this study, and the protocol was registered on PROSPERO with the number CRD-42023445886.

## Introduction

Hallux Abductus Valgus (HAV) was first described by Carl Hueter^1^ and is characterized as one of the most common foot deformities seen in clinical practice^2,3^. It typically presents with pain and is caused by abnormal biomechanics of the first metatarsophalangeal joint (MTPJ) during the propulsive phase of gait^4^. The hallux adopts a valgus position due to the action of the adductor muscle, resulting in lateral deviation of the proximal phalanx over the head of the first metatarsal. The first metatarsal becomes positioned in varus, leading to contracture of the lateral capsule and elongation of the medial capsule. While the transverse ligament keeps the sesamoids anchored to the second metatarsal, the first metatarsal laterally shifts and flattens its crest^1,5^. The cumulative effect of these movements includes progressive abduction and pronation of the first phalanx, adduction, pronation, and elevation of the first metatarsal, and lateral contracture of the capsular structure at the first MTPJ^2^.

Minimally invasive surgery (such as Reverdin Isham and Akin, Bosch and Minimally Invasive Chevron and Akin) represents a series of surgical techniques aimed at treating foot pathologies with minimal incisions, leading to definitive outcomes^6^. These techniques have exhibited remarkable efficacy in patient populations^7^. One notable advantage of these approaches is the elimination of the requirement for hemostasis, as controlled bleeding facilitates the removal of bone debris and mitigates the generation of heat during bone milling^8^. Moreover, these procedures are associated with minimally invasive techniques, owing to the utilization of small incisions that minimize damage to neighboring soft tissues, thereby resulting in accelerated recovery times^6^. The employment of intracapsular osteotomies contributes to favorable wound healing outcomes, while simultaneously reducing the duration of surgical intervention and the need for extensive instrumentation^9^. Furthermore, external fixation is limited, often accomplished through the utilization of compression bandages, thus obviating the need for osteosynthesis materials. Consequently, immediate weight-bearing is feasible with the aid of a rigid, flat-soled shoe^8,10^.

The chosen technique for the minimally invasive surgical treatment of moderate HAV is the Reverdin-Isham and Akin osteotomy. Reverdin Isham is a percutaneous surgical procedure without osteosynthesis^11^ in which an intracapsular wedge-shaped osteotomy is performed at the medial neck of the metatarsal bone, with a dorsal distal to plantar proximal angle of approximately 25°–45°^11–13^. In 1981, Isham refined the Reverdin technique initially described in 1881 for HAV by modifying the angulation of the osteotomy^14^. This technique achieves the realignment of the articular surface, corrects Distal Metatarsal Articular Angle (DMAA), improves the Hallux Abductus Valgus angle (HV_angle_), and stabilizes the forces at the head of the first metatarsophalangeal joint^15,16^. The intracapsular osteotomy becomes a highly stable procedure, obviating the need for internal fixation^12^. This surgery is indicated for patients with symptomatic medial bunion, with a normal range of motion at the first metatarsophalangeal joint without crepitus or degenerative changes. It is also recommended for congruent deviated joints with an intermetatarsal angle (IMA) of less than 20° for a straight foot and less than 16° for a foot in adduction with increased DMAA^12^.

HAV is a common foot deformity that can significantly impact a person's quality of life, causing pain, discomfort, and functional limitations. Understanding the effectiveness of Reverdin-Isham and Akin procedures is essential to guide healthcare professionals in making informed treatment decisions. Currently, there has been a notable increase in the number of case series published in the scientific literature such as Biz et al.^15^, Restuccia et al.^17^, Ribeiro et al.^13^, Severyns et al.^12^, Rodriguez-Reyes et al.^18^, Bauer et al.^11^, Bauer et al.^19^. Indeed, a meta-analysis conducted by Kaufmann et al.^20^ discussed a similar research question. However, these authors did not provide adequate reporting on critical aspects such as meta-regressions, and complications. By conducting this systematic review and meta-analysis, we can advance our understanding of the efficacy and safety of Reverdin-Isham and Akin surgical procedures, ultimately improving the care and outcomes for patients with moderate HAV. Therefore, the main objective of this systematic review and meta-analysis was to analyze the effect of Reverdin Isham and Akin surgical procedure on functional (i.e., The American Orthopedic Foot & Ankle Society [AOFAS scores]) and radiological (i.e., IMA, HV_angle_ and DMAA) in patients with moderate HAV. However, given the paramount importance of safety when performing surgical procedures, the second objective of this study was to provide a comprehensive assessment of the complications associated with these surgical procedures. Finally, meta-regressions were conducted to explore the relationship between baseline scores of the included outcomes and improvements achieved after the Reverdin surgical procedure.

## Methods

### Registry of systematic review protocol

This systematic review and meta-analysis was developed using the Reporting Items for Systematic Reviews and Meta-analysis (PRISMA) statement guidelines^21^. See Supplementary File 1. In addition, the Prisma in Exercise, Rehabilitation, Sport medicine and Sports science (PERSiT) was also implemented^22^. The protocol was pre-registered on PROSPERO.

### Eligibility criteria

To be included, studies had to adhere to the following criteria:

*Type of studies:* randomized and non-randomized controlled trials were included to evaluate the effect of the Reverdin-Isham and Akin surgical technique. Studies published in both Spanish and English languages were considered for analysis. Additionally, due to the nature of the surgical procedure, case reports were also included, allowing for the inclusion of studies where all available data was reported. *Type of participant:* participants needed to be above 18 years of age and exhibit a moderate/severe degree of HAV. Patients with moderate hallux valgus display a hallux abductus angle ranging between 20° and 40°, and a first intermetatarsal angle of 8° to 15°. In the case of severe hallux valgus, these values exceed those observed in moderate hallux valgus^23^.

*Types of interventions:* the interventions had to include exostectomies, Reverdin-Isham osteotomy, Akin osteotomy, tenotomy of adductus tendon and lateral capsulotomy. *Type of outcome measures:* the outcomes of interest were AOFAS scores, IMA, DMAA and, HV_angle_.

### Search strategy

The primary search focused on studies reporting the effect Reverdin Isham and Akin surgical technique on functional and radiological outcomes. The final search date July 18^th^, 2023. Searches were performed through MEDLINE via PubMed and Web of Science. A PICO strategy was used to build search criteria for electronic databases. The PICO consisted of terms for Reverdin Isham and Akin surgical technique on functional (i.e., AOFAS scores) and radiological outcomes (i.e., IMA, DMAA and HV_angle_). The search string used for MEDLINE/PubMed was: ("Minimally Invasive Surgical Procedures"[MeSH Terms] OR ("reverdin isham"[Title/Abstract] OR "reverdin isham osteotomy"[Title/Abstract] OR "reverdin isham percutaneous"[Title/Abstract] OR "reverdin isham percutaneous osteotomy"[Title/Abstract] OR "reverdin isham procedure"[Title/Abstract])) AND ("Podiatry"[MeSH Terms] OR "Hallux Valgus"[MeSH Terms] OR ("Podiatry"[Title/Abstract] OR "Hallux Valgus"[Title/Abstract])). The searches strings used for other databases were adapted using Polyglot Search Translator Tool (https://sr-accelerator.com/#/polyglot) ^24^.These searches strings are reported in Supplementary File 2.

### Study selection

To remove duplicate references, an online tool (https://www.sr-accelerator.com/#/deduplicator) was used first, followed by manual removal using the Mendeley reference manager. Two authors (Author 1 and Author 4) independently reviewed titles and abstracts for initial eligibility using an online tool (https://www.sr-accelerator.com/#/disputatron). Any disagreements were resolved through discussion, and if necessary, the third reviewer (Author 4) was consulted.

### Study coding and data extraction

All data extraction was made independently by two authors (Author 1 and Author 2). From the included studies, the following data was extracted and coded: (1) authors, year of publication, (2) the number of participants in the study and characteristics such as sex, age, body mass, body mass index, height, and (3) follow-up. Mean and standard deviation of AOFAS scores as well as mean and standard deviation of radiological outcomes (i.e., IMA, HV_angle_ and DMAA) were collected in a single Excel spreadsheet. In addition, number of surgeon, partial and/or absolute reliability scores were also obtained.

### Methodological quality and risk of *bias*

Two researchers (Author 1 and Author 5) independently assessed the Joanna Briggs Institute (JBI) critical appraisal tool for case series studies. This tool includes 10 questions addressing the internal validity and risk of bias of case series designs, particularly confounding, selection, and information bias, in addition to the importance of clear reporting^25^. A complete description of this tool as well as the GRADE system is found in Supplementary Table 1 (Supplementary File 3).

### Statistical analysis

The sample size, and means, standard deviation, 95% confident intervals (CI_95%_) for AOFAS scores, as well as radiological outcomes were extracted independently by two authors (Author 1 and Author 5). The effect size calculations (i.e., standardized mean differences [SMD] and mean differences [MD]) as well as meta-analytical statistical procedures is explained in Supplementary File 4. Briefly, a random-effects meta-analysis was performed for each separate outcome using Hartung-Knapp/Sidik-Jakman adjustment to calculate model parameters for small-sample correction^26^.

### Ethical approval and consent to participate

This study design and protocol were performed in accordance with the PRISMA Statement. The protocol was registered previously on PROSPERO CRD-42023445886.

## Results

### Search results

**Figure 1:**
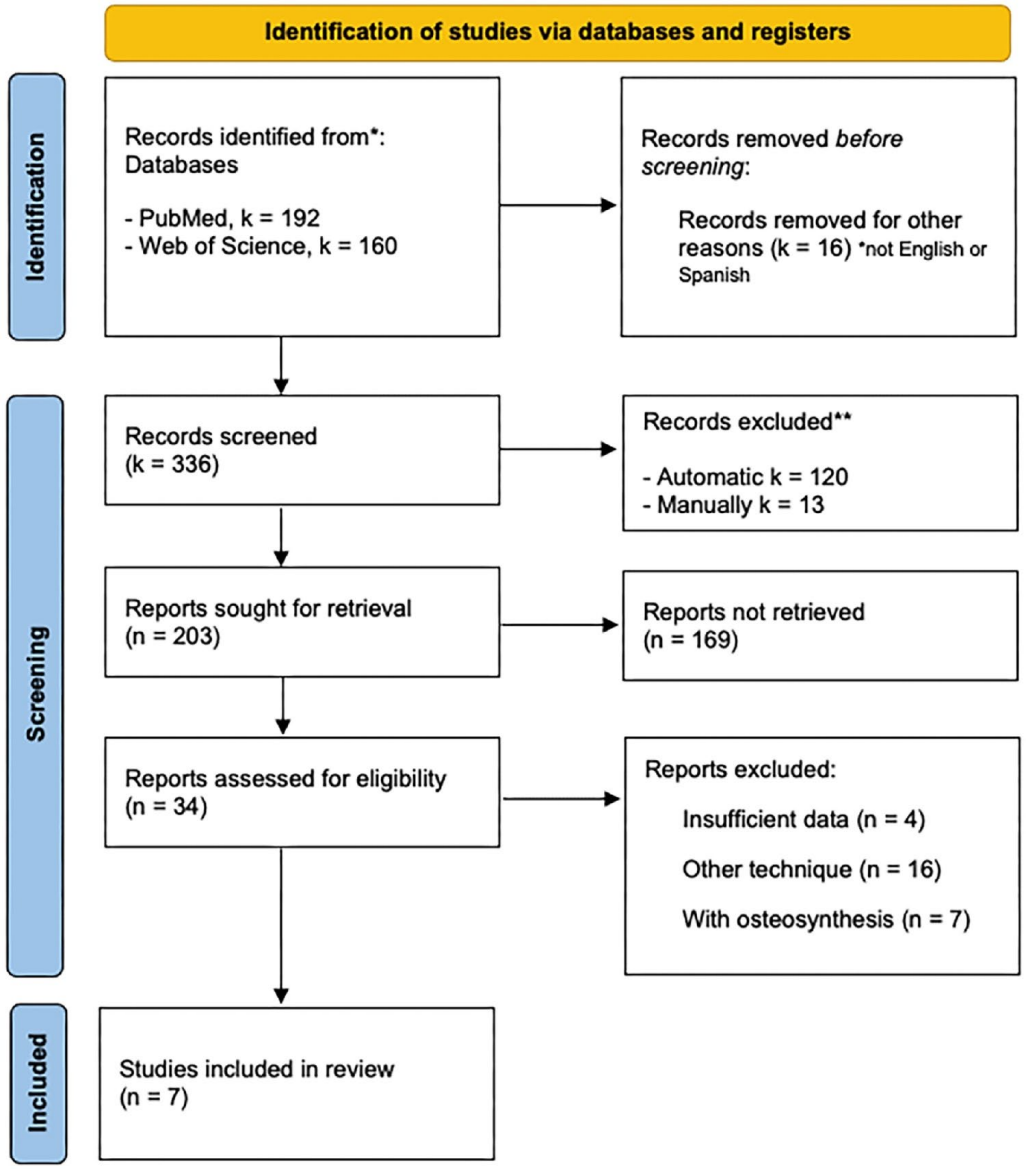
PRISMA flow diagram chart.

Figure 1 presents the flow chart detailing the various stages of the literature search and the selection of studies included in this review. The initial search of electronic databases yielded 352 records. Before the screening process, a total of 16 studies were removed due to filters set for journal articles and those not written in English or Spanish, dated. Duplicate studies were then removed (k = 120 through automated tool and 13 manually), after which an additional 169 studies were excluded based on the title and abstract screening. Furthermore, 27 studies were excluded after full-text assessment (see Supplementary File 5). A total of 7 studies (Biz et al.^15^, Restuccia et al.^17^, Ribeiro et al.^13^, Severyns et al.^12^, Rodriguez-Reyes et al.^18^, Bauer et al.^11^, Bauer et al.^19^. Indeed, a meta-analysis conducted by Kaufmann et al.^20^ were therefore included.

### Participants and interventions characteristics

The cumulative sample size across all studies was 554 patients, encompassing 643 operated feet. 78 were males and 298 were females. Only one study reported body mass. Information regarding the number of surgeons was provided by merely two studies; in the Biz et al.^15^ study, all surgeries were conducted by a single surgeon, whereas the surgeries in the Ribeiro et al.^13^ study were performed by two surgeons. More information can be found in Supplementary Table 2 (Supplementary File 6).

### Risk of *bias*

Table 1 summarized the JBI appraisal tool scores for selected studies. From a quantitative point of view, item 1 was label as “Yes” in k = 6 (86%), “unclear” in k = 1 (14%) and “No” in k = 0 (0%). Item 2 was label as “Yes” in k = 4 (57%), “unclear” in k = 2 (29%) and “No” in k = 1 (14%). Item 3 was label as “Yes” in k = 5 (71%), “unclear” in k = 2 (29%) and “No” in k = 0 (0%). Item 4 was label as “Yes” in k = 3 (43%), “unclear” in k = 4 (57%) and “No” in k = 0 (0%). Item 5 was label as “Yes” in k = 6 (86%), “unclear” in k = 1 (14%) and “No” in k =  = 0 (0%). Item 6 was label as “No” in k = 7 (100%). Item 7 was label as “Yes” in k = 4 (57%), “unclear” in k = 0 (0%) and “No” in k = 3 (43%). Item 8 was label as “Yes” in k = 6 (86%), “unclear” in k = 0 (0%) and “No” in k = 1 (14%). Item 9 was label as “Yes” in k = 5 (71%), “unclear” in k = 0 (0%) and “No” in k = 2 (29%). Item 10 was label as “Yes” in k = 4 (57%), “unclear” in k = 3 (43%) and “No” in k = 0 (0%).

**Table 1:**
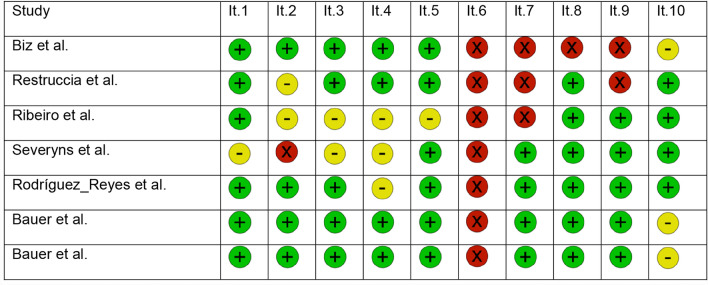
Results for Joanna Briggs Institute (JBI) critical appraisal tool for case series studies.

it. 1 = Were there clear criteria for inclusion in the case series?, it. 2 = Was the condition measured in a standard, reliable way for all participants included in the case series?, it. 3 = Were valid methods used for identification of the condition for all participants included in the case series?, it. 4 = Did the case series have consecutive inclusion of participants?, it. 5 = Did the case series have complete inclusion of participants?, it. 6 = Was there clear reporting of the demographics of the participants in the study?, it. 7 = Was there clear reporting of clinical information of the participants?, it. 8 = Were the outcomes or follow-up results of cases clearly reported?, it. 9 = Was there clear reporting of the presenting sites’/clinics’ demographic information?, and it. 10 = Was statistical analysis appropriate?. "Yes" was represented by the color green, "Unclear" by yellow, and "No" by red.

### Meta-analysis results

**Figure 2:**
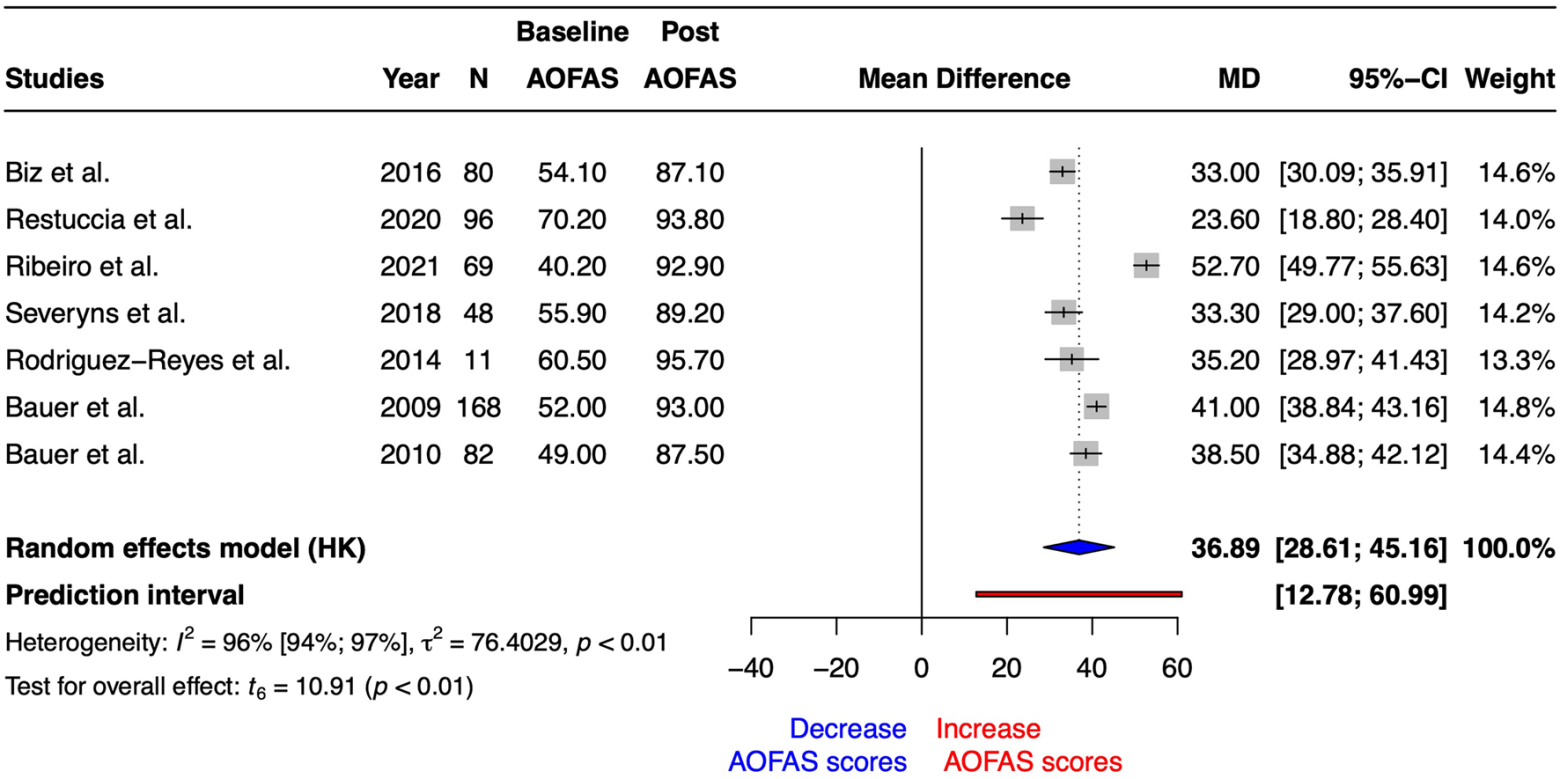
Forest plot for American Orthopedic Foot & Ankle Society (AOFAS) scores after Reverdin surgical procedure. N = total sample size, MD = mean differences, 95%CI = 95% confident interval.

After Reverdin surgical procedure the univariate meta-analysis revealed a statistically significant increase on AOFAS scores (SMD = 3.29 [1.66–4.92], t_6_ = 4.94, *p* = 0.003 and MD = 36.89 [28.61–45.16], t_6_ = 10.91, *p* < 0.0001). The heterogeneity obtained correspond to I^2^ = 96% (94–98%). On the other hand, prediction interval ranged from 13 to 61. Figure 2 summarized the forest plot for AOFAS scores MD. Risk of bias and GRADE are summarized in Tables 1 and 3, respectively.

**Figure 3:**
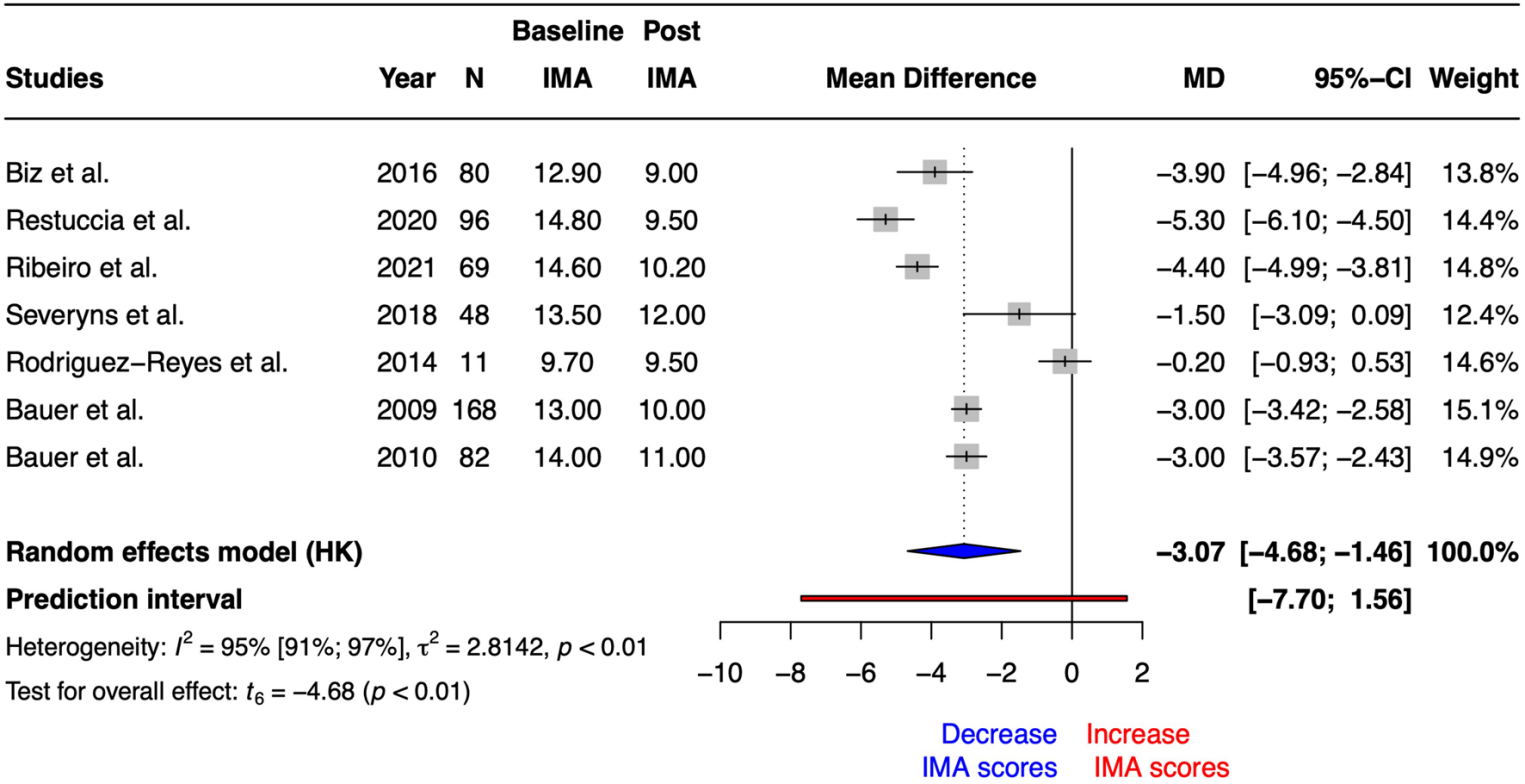
Forest plot for Intermetatarsal Angle (IMA) scores after Reverdin surgical procedure.

Regarding IMA, the univariate meta-analysis revealed a statistically significant increase after Reverdin intervention (SMD = − 1.06 [− 1.55 to − 0.58], t_6_ = 5.40, *p* = 0.002 and MD = − 3.07° [− 4.68 to − 1.46], t_6_ = − 4.68, *p* = 0.003). The heterogeneity obtained correspond to I^2^ = 95% (91% to 97%). On the other hand, prediction interval ranged from − 8 to 1.56. Figure 3 summarized the forest plot for IMA scores MD. Risk of bias and GRADE are summarized in Tables 1 and 3, respectively.

### ***Results were expressed in degrees (***°***)***

**Figure 4:**
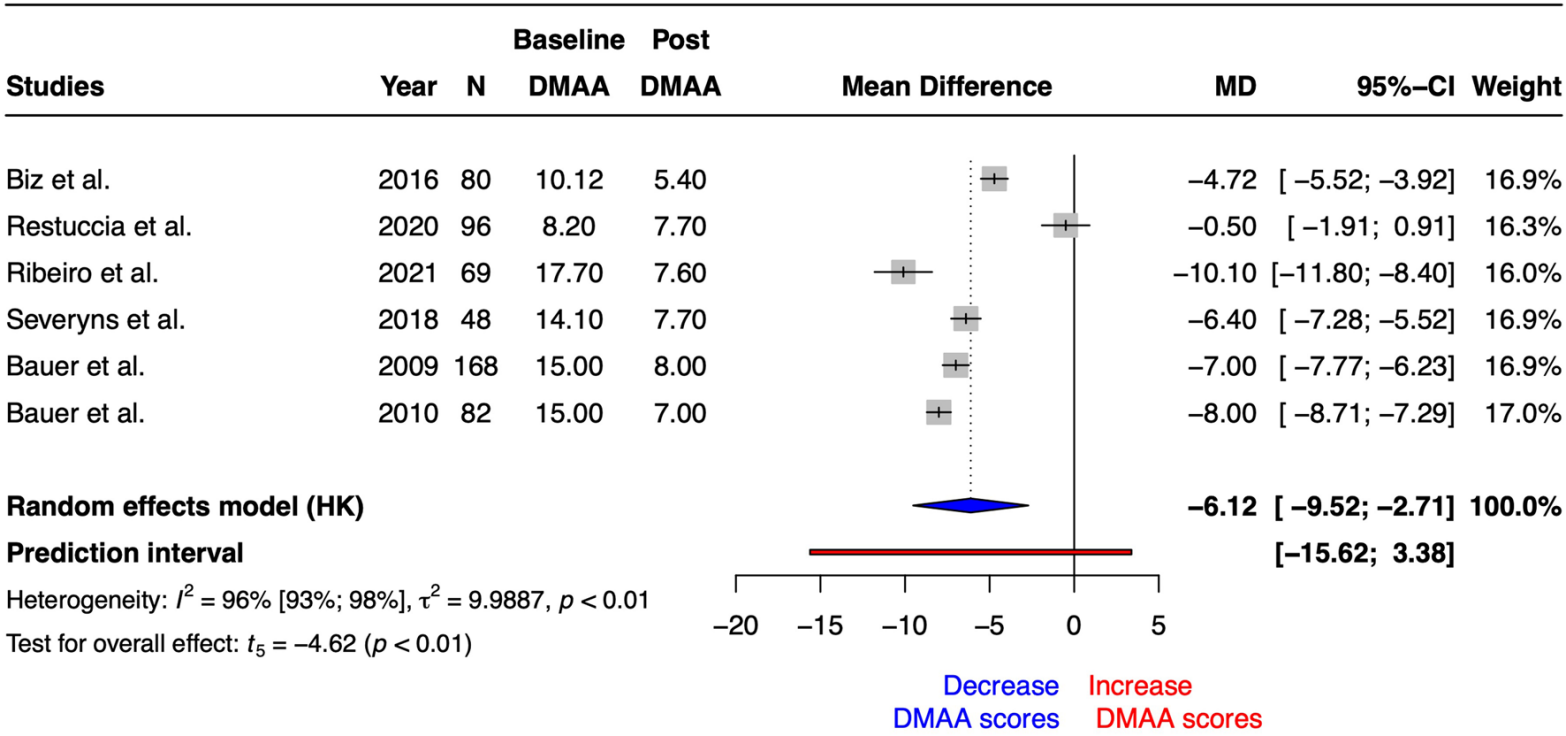
Forest plot for Distal Metatarsal Articular Angle (DMAA) scores after Reverdin surgical procedure. N = total sample size, MD = mean differences, 95% CI = 95% confident interval. Results were expressed in degrees (°).

In relation to DMAA, the univariate meta-analysis demonstrated a statistically significant increase following the Reverdin intervention (SMD = − 1.05 [− 1.62 to − 0.47], t_5_ = 4.69, *p* = 0.005 and MD = − 6.12 [− 9.52 to − 2.71], t_5_ = − 4.62, *p* = 0.006). The heterogeneity obtained correspond to I^2^ = 96% (93–98%). On the other hand, prediction interval ranged from − 16 to 3. Figure 4 summarized the forest plot for DMAA scores MD. Risk of bias and GRADE are summarized in Tables 1 and 3, respectively.

**Figure 5:**
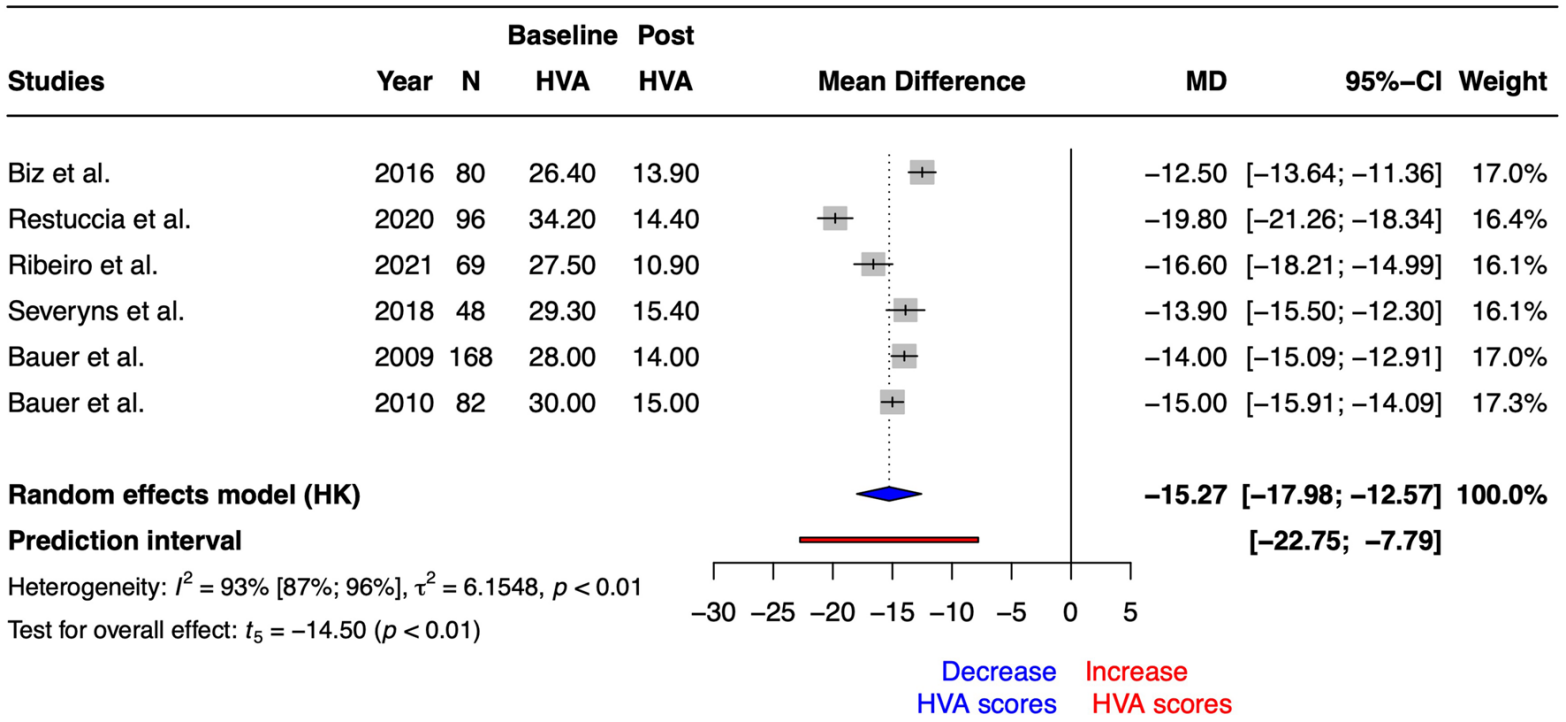
Forest plot for Hallux Valgus Angle (HV_angle_) scores after Reverdin surgical procedure. N = total sample size, MD = mean differences, 95%CI = 95% confident interval. Results were expressed in degrees (°).

Regarding HV_angle_, the univariate meta-analysis revealed a statistically significant increase after Reverdin intervention (SMD = − 2.05 [− 2.54 to − 1.57], t_5_ = − 10.88, *p* = 0.0001 and MD = − 15.27 [− 17.98 to − 12.57], t_5_ = − 14.50, *p* < 0.0001). The heterogeneity obtained correspond to I^2^ = 93% (87–96%). On the other hand, prediction interval ranged from − 23 to 8. Figure 5 summarized the forest plot for DMAA scores MD. Risk of bias and GRADE are summarized in Tables 1 and 3, respectively.

## Complications

The studies included in the present systematic review and meta-analysis have reported the following complications associated with the Reverdin surgical technique. In the study conducted by Biz et al.^15^, a total of 25 patients encountered complications, out of which 6 (24%) patients experienced serious complications, such as recurrence and severe stiffness. The remaining 19 (76%) patients dealt with minor complications like slight loss of normal range of motion in the MTP joint and delayed wound healing. On the contrary, Retuccia et al.^17^ did not report any major complications. However, among their 25 patients, minor complications were noted, including incomplete correction of deformities, transfer metatarsalgia, and HV overcorrection. Ribeiro et al.^13^ documented complications in 8 (25%) patients, with 2 having serious complications, namely recurrence and third metatarsal stress fracture, while 6 (75%) faced minor complications like Hallux hypoesthesia, transfer metatarsalgia, and type 1 complex regional pain syndrome. Severyns et al.^12^ identified complications in 15 (7%) patients, including one case of deep vein thrombosis and 14 (93%) minor complications like transfer metatarsalgia, delayed cutaneous healing, recurrences, and hallux hypoesthesia. In Bauer et al.^19^ study, 10 patients experienced complications, with 5 (50%) facing serious complications such as deep vein thrombosis and stiffness of the first MTP joint, while the other 5 (50%) developed type 2 complex regional pain syndrome. In another study by Bauer et al.^11^, 24 complications were reported, with 14 (58%) being major complications like fractures, stiffness of the first MTP joint, and recurrences, while 10 (42%) were minor complications such as DMAA overcorrection, complex regional pain syndrome, and transfer metatarsalgia. Lastly, the article by Rodriguez-Reyes et al.^18^ did not provide any information about complications.

### Meta-regressions

**Figure 6:**
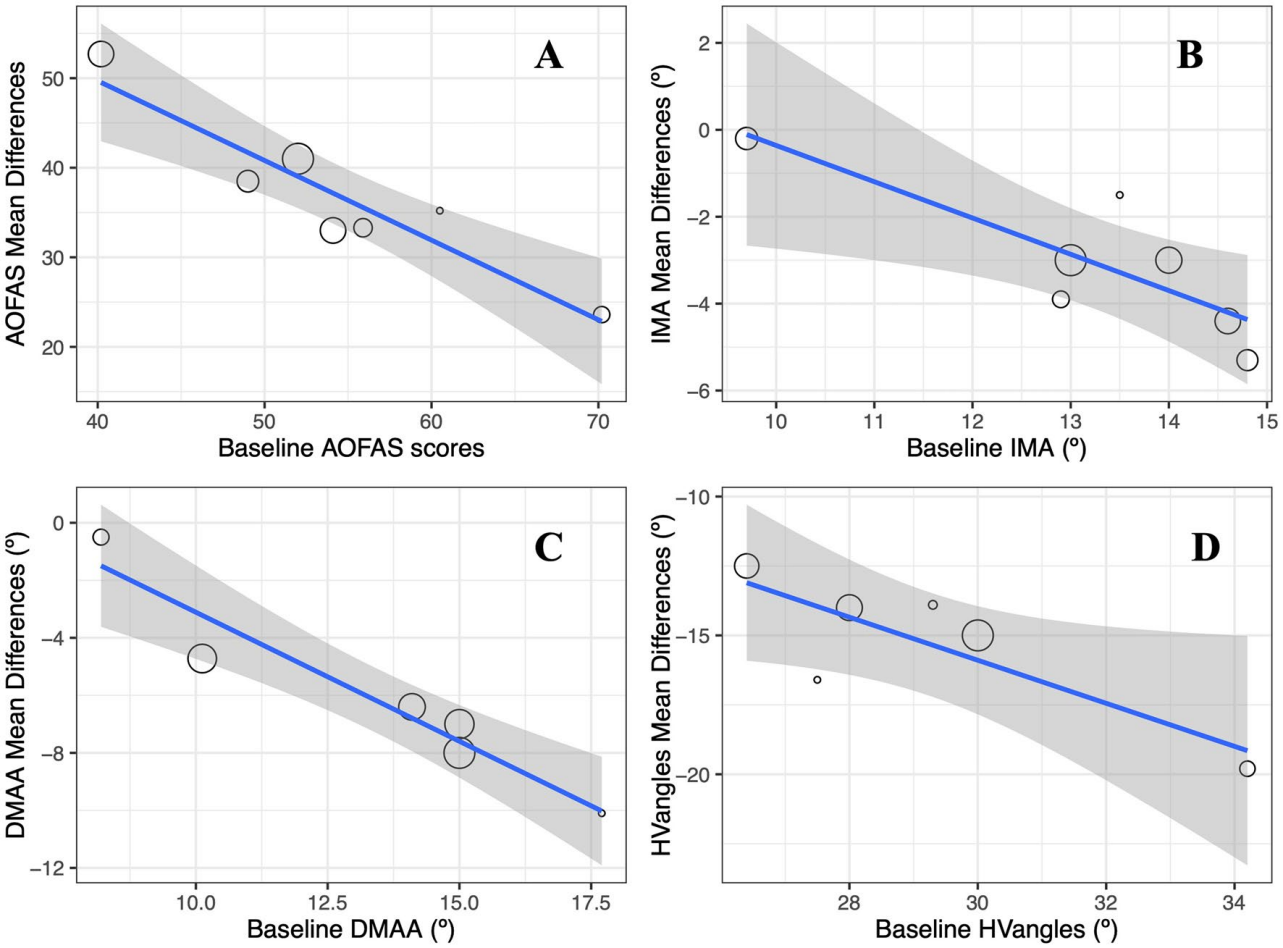
The meta-regression results were obtained after analyzing the relationship between baseline scores and improvement following the Reverdin surgical procedure. (**A**) AOFAS vs. AOFAS baseline, (**B**) IMA versus IMA baseline, (C*) DMAA versus DMAA baseline and, (**D**) HV_angle_ versus HV_angle_ baseline. The blue line represents the regression line, while the grey shaded areas denote the 95% confidence intervals.

The improvements observed in all outcomes included in this meta-analysis were found to be associated with the baseline scores of each variable. The results obtained from the analysis are presented and described in Table 2 and Fig. 6.

**Table 2:** Meta-regression Results from Mean Baseline Scores (American Orthopedic Foot & Ankle Society [AOFAS], Intermetatarsal Angle [IMA], Distal Metatarsal Articular Angle [DMAA], and Hallux Valgus Angle [HV_angle_]) and Improvement Achieved.

Outcome	Estimate	SE	z-value	95% CI	*p*-value	R^2^ (%)
Intercept	85.70	8.42	10.18	69 to 102	< 0.0001	87
AOFAS BL	− 0.90	0.15	− 5.86	− 1.19 to − 0.60	< 0.0001	
Intercept	8.04	3.39	2.37	1.40 to 14.69	0.018	64
IMA BL	− 0.84	0.25	− 3.31	− 1.34 to − 0.34	0.001	
Intercept	5.47	2.06	2.65	1.42 to 9.50	0.008	91
DMAA BL	− 0.87	0.15	− 5.77	− 1.17 to − 0.57	< 0.0001	
Intercept	7.59	8.16	0.93	− 8.40 to 23.59	0.352	60
HVangle BL	− 0.78	0.28	− 2.81	− 1.33 to − 0.24	0.005	

*AOFAS BL* American Orthopaedic Foot & Ankle Society baseline, *IMA BL* Intermetatarsal Angle baseline, *DMAA BL* Distal Metatarsal Articular Angle baseline, *HV*_*angle*_* BL* Hallux Valgus Angle baseline, *95% CI* 95% Confident interval, *SE* Standard error, *R*^2^ Percentage of explained variance.

**Figure 7:**
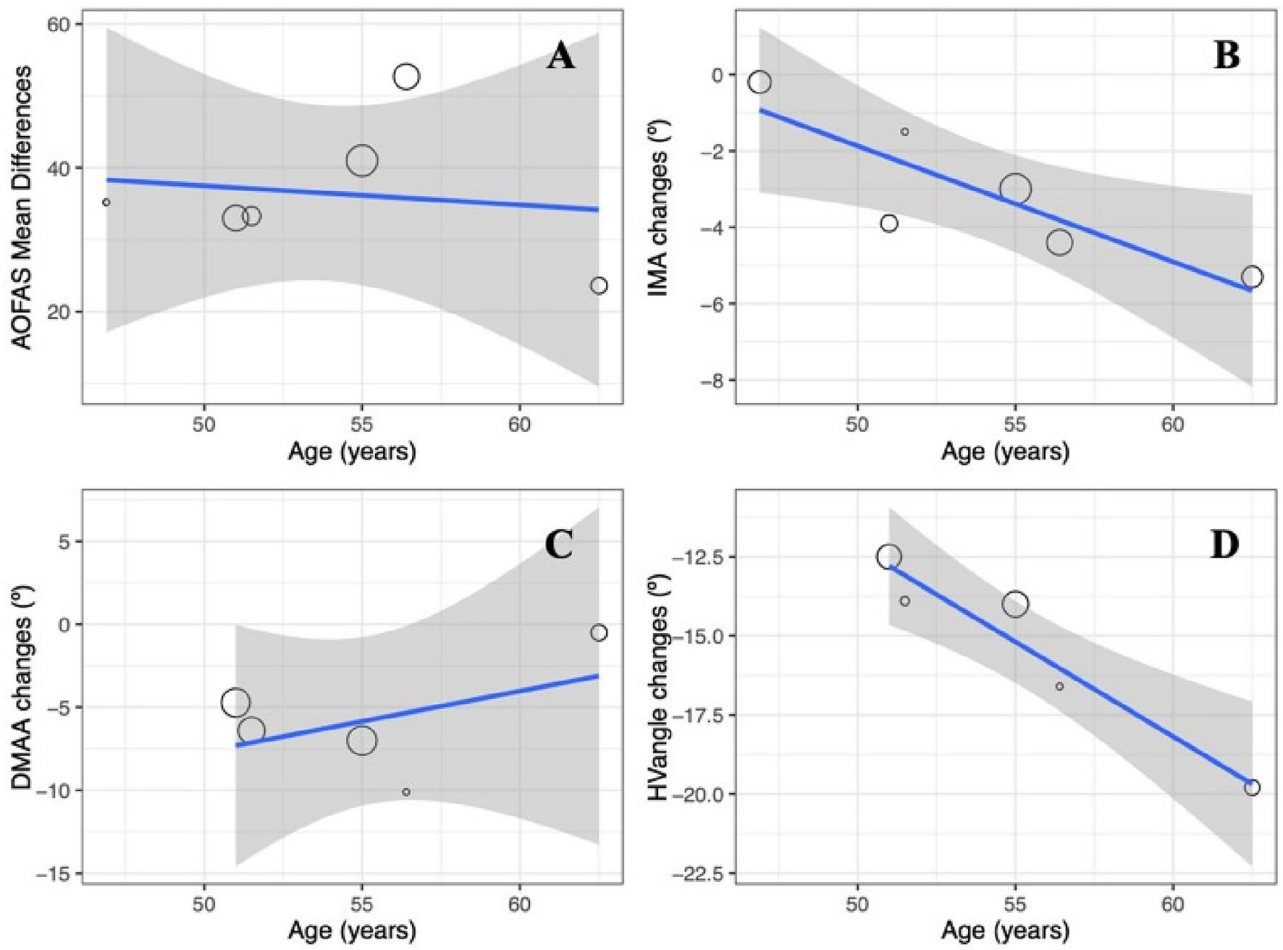
The meta-regression results were obtained after analyzing the relationship between age and improvement following the Reverdin surgical procedure. (**A**) AOFAS versus age, (**B**) IMA versus age, (C*) DMAA versus age and, (**D**) HV_angle_ versus age. The blue line represents the regression line, while the grey shaded areas denote the 95% confidence intervals.

The results of the meta-regressions, Fig. 7, which examined the relationship between age and improvements in outcomes (i.e., AOFAS, IMA, DMAA, and HV_angle_), were as follows: The associations between AOFAS and age, as well as DMAA and age, did not exhibit statistical significance (estimate = − 0.26, *p* = 0.768 and estimate = 0.36, *p* = 0.339, respectively). However, statistically significant relationships were identified when comparing age with IMA (estimate = − 0.31, *p* = 0.0003, R^2^ = 81%) and with HV_angle_ (estimate = − 0.60, *p* < 0.0001, R^2^ = 97%).

### GRADE system

The GRADE system for AOFAS, IMA, DMAA and HV_angle_ is summarized in Table 3.

**Table 3:** The Grading of Recommendations, Assessment, Development, and Evaluation (GRADE) system was utilized to assess the quality and strength of the included outcomes (i.e., American Orthopedic Foot & Ankle Society [AOFAS] scores, Intermetatarsal Angle [IMA], Distal Metatarsal Articular Angle [DMAA], and Hallux Valgus Angle [HV_angle_]) in the systematic review and meta-analysis.

Outcome	Summary of findings						Quality of evidence synthesis (GRADE)	
	k	n	Effect (95% CI)	Direction effect compared to baseline	Imprecision	Inconsistency	Risk of bias	Overall quality
AOFAS scores								
AOFAS scores	7	554	3.29 (1.66–4.92)	↑	None	− 1	− 1	●○○○○ Very Low
IMA scores								
IMA scores	7	554	− 1.06 (− 1.55 to − 0.58)	↑	None	− 1	− 1	●○○○○ Very Low
DMAA scores								
DMAA scores	6	543	− 1.05 (− 1.62 to − 0.47)	↑	None	− 1	− 1	●○○○○ Very Low
HV_angle_								
HV_angle_	6	543	− 2.05 (− 2.54 to − 1.57)	↑	None	− 1	− 1	●○○○○ Very Low

*AOFAS BL* American Orthopedic Foot & Ankle Society, *IMA* Intermetatarsal Angle, *DMAA* Distal Metatarsal Articular Angle, *HV*_*angle*_* BL* Hallux Valgus Angle, *k* number of studies, *n* number of feet.

### Publication bias

**Figure 8:**
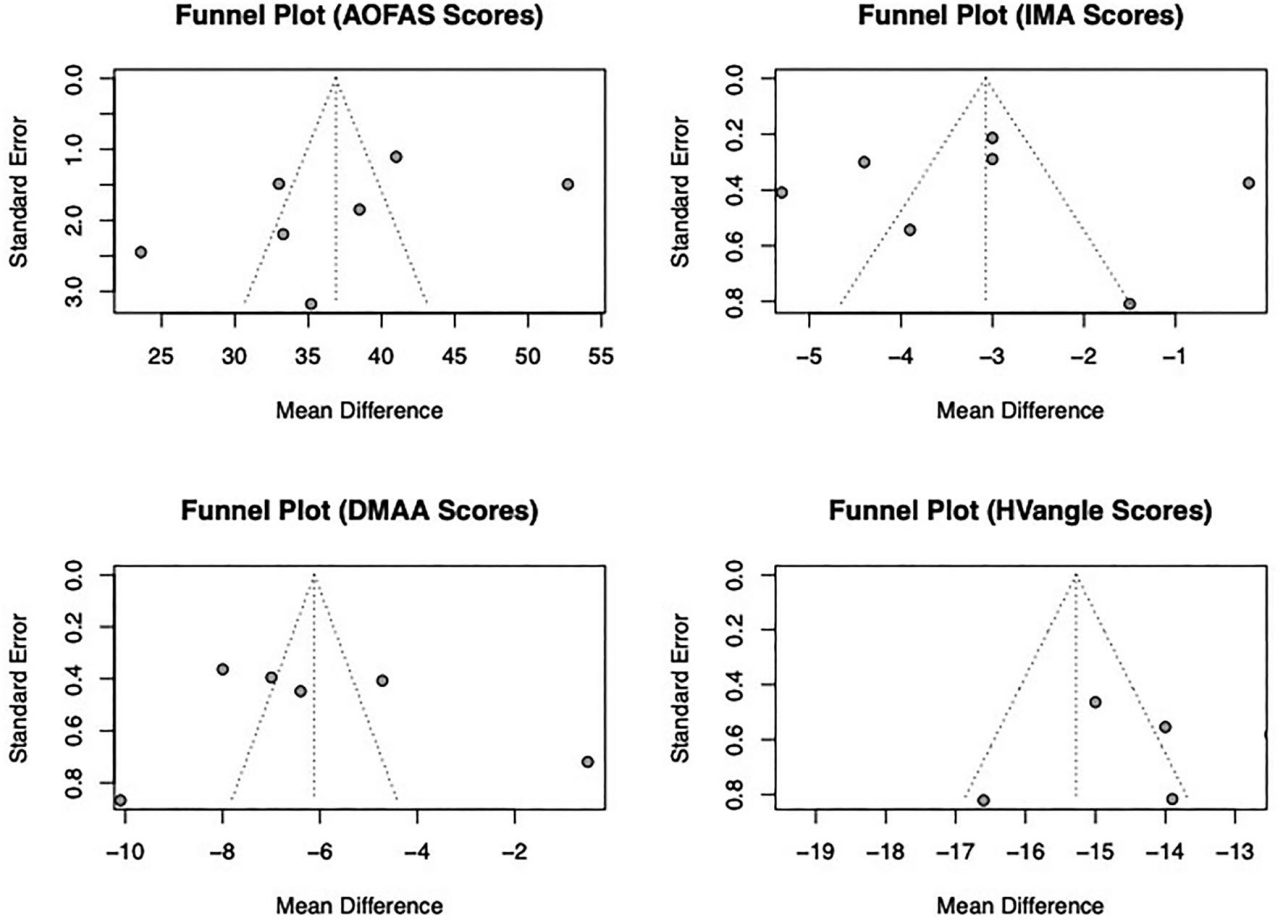
Funnel Plot for American Orthopedic Foot & Ankle Society (AOFAS) scores, Intermetatarsal Angle (IMA), Distal Metatarsal Articular Angle (DMAA), and Hallux Valgus Angle (HV_angle_).

No publication bias was detected both from a visual point of view (i.e., funnel plots, see Fig. 8) and statistical point of view (i.e., egger test).

The results obtained for egger tests for AOFAS, IMA, DMAA and HV_angle_ are summarized in Table 4.

**Table 4:** Results from Egger test for American Orthopedic Foot & Ankle Society (AOFAS) scores, Intermetatarsal Angle (IMA), Distal Metatarsal Articular Angle (DMAA), and Hallux Valgus Angle (HV_angle_).

Outcome	Intercept	95% CI	t-value	*p*-value
AOFAS	− 6.96	− 18.62 to 4.7	− 1.69	0.295
IMA	0.62	− 9.41 to 10.65	0.12	0.908
DMAA	4.04	− 11.6 to 19.67	0.51	0.639
HV_angle_	− 6.25	− 20.49 to 8	− 0.86	0.438

*AOFAS* American Orthopedic Foot & Ankle Society, *IMA BL* Intermetatarsal Angle, *DMAA* Distal Metatarsal Articular Angle, *HV*_*angle*_* BL* Hallux Valgus Angle, *95% CI* 95% Confident interval.

## Discussion

The primary aim of this systematic review and meta-analysis was to examine the impact of the Reverdin-Isham and Akin surgical procedures on functional and radiological outcomes in patients diagnosed with moderate HAV. The main findings indicated that the Reverdin and Akin surgical technique was a safe and beneficial technique for addressing moderate HAV. The functional assessment, as measured by AOFAS scores, exhibited a significant average improvement of 36 points, indicating enhanced foot function and reduced pain for patients. Moreover, notable improvements were observed in radiological outcomes, with reductions of − 3.07°,− 6.12°, and − 15.27° observed in IMA, DMAA, and HV_angle_, respectively. On the other hand, the primary complications reported in the included studies were recurrence, deep vein thrombosis and fracture. Finally, a substantial, statistically significant, and negative relationship was observed between the baseline scores and the improvements following the Reverdin surgical procedure. That is, patients with poorer scores in both functional and radiological outcomes demonstrated a more favorable response in the analyzed outcomes after surgical procedure. These findings underscore the effectiveness of the Reverdin-Isham and Akin surgical technique in improving both functional and radiological parameters in patients with moderate HAV. However, these results come from studies with some concerns or high risk of bias. Overall, the study highlights the potential benefits of these surgical interventions in enhancing patient outcomes and provides valuable insights for healthcare professionals when considering treatment options for moderate HAV.

The AOFAS scale is recognized as a valuable instrument for outcome assessment in numerous studies^18,27^. However, in this meta-analysis, although we found values ranging from 0 out to 100 and represented as pre-post mean differences, we did not find any studies that breakdown each aspect of the scale in the same manner as specified by Naranjo-Ruiz et al.^28^, i.e., representing the scores obtained on each dimension. This limitation hinders the individual analysis of critical variables such as pain, function, and alignment, which could provide new perspectives in evaluating each surgical intervention.

Several studies have compared functional outcomes after Reverdin osteotomy versus Chevron osteotomy for hallux valgus, using the AOFAS scale. A study by Kaufmann et al.^20^, with 49 patients (59 feet), found a notably greater improvement in AOFAS scores after Reverdin osteotomy (increasing from 48 to 91 points) compared to Chevron osteotomy (improving from 65 to 95 points). These findings align with our results; however, in our meta-analysis, we identified an improvement exceeding 36 points. Interestingly, meta-regression results showed a negative and statistically significant relationship between baseline AOFAS scores and the AOFAS effect size (see Table 2). That is, the range of improvement of those patients with lower AOFAS scores will be larger than those patients with higher values. Nevertheless, the GRADE system provided a very low overall quality for AOFAS, mainly due to inconsistency and risk of bias in the included studies.

The Reverdin technique has demonstrated significant improvements in radiological angles like the IMA, HVangle, and DMAA. On average, reductions of − 3.07° (ranging from − 4.68° to − 1.46°) for the IMA angle, − 15.27° (ranging from − 17.98° to − 12.57°) for the HVangle, and − 6.12° (ranging from − 9.52° to − 2.71°) for the DMAA angle were observed. Additionally, the Reverdin technique is characterized as a minimally invasive procedure with a short recovery duration and low risk of complications. Notably, the meta-regression results revealed negative and statistically significant relationships between baseline angle scores and the corresponding effect sizes after the Reverdin surgery. Patients with higher initial HVangle and IMA angle values experienced greater improvements in correcting these radiological parameters compared to those with lower baseline angles. These findings suggest that the Reverdin osteotomy may be particularly effective for patients with more severe deformities prior to the surgical intervention, enabling substantial correction of intermetatarsal and hallux valgus angles.

The risk of bias analysis highlights specific issues with the standardization of procedures for collecting and reporting results in the field of orthopedics, particularly regarding items 6 and 7. These items pertain to the reporting of participant demographics and clinical information in studies. The lack of standardized procedures for these aspects can lead to data inconsistencies, which in turn might affect the validity and reproducibility of the studies. This is critical as demographics and clinical information are fundamental to understanding the context and applicability of study results to broader populations. The non-standardization in these areas risks undermining confidence in the findings and limits the ability to generalize results, potentially biasing the interpretations and conclusions drawn from such studies.

The meta-regression results from our study revealed significant associations between baseline scores and improvements in IMA, DMAA, and HVangle after Reverdin surgery (see Fig. 6), consistent with the literature^11,15,19^. The meta-analysis identified varying effects of age on the enhancement of different measurements after minimally invasive surgery. There was no statistically significant impact of age on the improvement in clinical AOFAS scores or DMAA angle after surgery, suggesting that changes in these measures are independent of age.

Several limitations might be considered when interpreting the results obtained in this meta-analysis. Firstly, from a methodological point of view, the absence of control groups in the included studies was a notable limitation. While the obtained effect sizes are promising and provide a general indication of the potential outcomes following the application of the Reverdin surgical technique, it is crucial to acknowledge that various confounding factors may influence the observed results. Nevertheless, in situations where there is a limited availability of high-quality experimental studies assessing effectiveness, case series may serve as the most valuable evidence to guide clinical practice^25^. Furthermore, it is worthing to highlight that the heterogeneity scores obtained in this meta-analysis were high. The presence of substantial heterogeneity suggests that there are differences among the included studies in terms of populations, or other relevant factors (such as the number of surgeons or the specific procedures during rehabilitation or estimated data from original studies such as Pearson correlations coefficients or SD´s). This heterogeneity may impact the overall validity and generalizability of the findings. Therefore, caution should be exercised when interpreting the results, and further research is needed to explore and address the sources of heterogeneity to obtain more robust and reliable conclusions. The absence of meta-regression or subgroup analysis (apart from baseline scores or age) in this study was attributed to the lack of homogeneity in terms of patient characteristics across the included studies. For future studies, it is recommended to systematically report information regarding patient characteristics, such as body mass, number of surgeons, exercise practices, or post-surgical procedure protocols. Including these details in a more standardized manner would enhance the comprehensiveness and reliability of the research findings, allowing for a more thorough analysis of the potential impact of these factors on outcomes.

## Conclusions

In conclusion, although the studies included in the review exhibit some degree of bias risk, the findings suggest that the Reverdin and Akin techniques are safe and effective in improving both functional and radiological parameters in patients with moderate to severe hallux valgus. Furthermore, the negative relationship observed between baseline factors and post-surgical improvements underscores the efficacy of these surgical techniques. This implies that patients with poorer initial conditions tend to experience more significant improvements following surgery, reaffirming the importance of considering these procedures as a valid and potentially transformative option for treating hallux valgus.

### Supplementary Information

Supplementary Information 1.

Supplementary Information 2.

Supplementary Information 3.

Supplementary Information 4.

Supplementary Information 5.

Supplementary Information 6.

## Data Availability

The data presented in this study are available upon request to the corresponding author.
