# Supplementary material for: Radiological and functional outcomes of Reverdin Isham osteotomy in moderate Hallux Valgus: a systematic review and meta-analysis
Source: Sci Rep. 2024 Jun 26;14:14781. doi: 10.1038/s41598-024-65440-3 (PMC11208448; doi:10.1038/s41598-024-65440-3)
Supplement: Supplementary file 2 — Supplementary Information 2. [file 41598_2024_65440_MOESM2_ESM.pdf]

Supplementary File 2. Searches strings for electronic databases.

The original search string was created in PubMed. The searches strings employed for Web of Science and MEDLINE via PubMed were translated using an automatic online tool (www.sr-accelerator.com/Polyglot):

1. **Search strategy for PubMed**

URL: https://pubmed.ncbi.nlm.nih.gov
Filters: no filters were applied.

("Minimally Invasive Surgical Procedures"[MeSH Terms] OR ("reverdin isham"[Title/Abstract] OR "reverdin isham osteotomy"[Title/Abstract] OR "reverdin isham percutaneous"[Title/Abstract] OR "reverdin isham percutaneous osteotomy"[Title/Abstract] OR "reverdin isham procedure"[Title/Abstract])) AND ("Podiatry"[MeSH Terms] OR "Hallux Valgus"[MeSH Terms] OR ("Podiatry"[Title/Abstract] OR "Hallux Valgus"[Title/Abstract])).

2. **Search strategy for Web of Science**

URL: https://www.webofscience.com/wos/alldb/basic-search
Filters: no filters were applied.

("Minimally Invasive Surgical Procedures" OR ("reverdin isham" OR "reverdin isham osteotomy" OR "reverdin isham percutaneous" OR "reverdin isham percutaneous osteotomy" OR "reverdin isham procedure")) AND (Podiatry OR "Hallux Valgus" OR (Podiatry OR "Hallux Valgus"))
