# Supplementary material for: Radiological and functional outcomes of Reverdin Isham osteotomy in moderate Hallux Valgus: a systematic review and meta-analysis
Source: Sci Rep. 2024 Jun 26;14:14781. doi: 10.1038/s41598-024-65440-3 (PMC11208448; doi:10.1038/s41598-024-65440-3)
Supplement: Supplementary file 3 — Supplementary Information 3. [file 41598_2024_65440_MOESM3_ESM.pdf]

Supplementary file 3. Risk of Bias and Grading of Recommendation Assessment, Development and Evaluation system.

The Joanna Briggs Institute (JBI) critical appraisal tool for case series studies. This tool includes 10 questions addressing the internal validity and risk of bias of case series designs, particularly confounding, selection, and information bias, in addition to the importance of clear reporting. The questions addressed in this scale were it. 1= Were there clear criteria for inclusion in the case series?, it. 2= Was the condition measured in a standard, reliable way for all participants included in the case series?, it. 3= Were valid methods used for identification of the condition for all participants included in the case series?, it. 4= Did the case series have consecutive inclusion of participants?, it. 5= Did the case series have complete inclusion of participants?, it. 6= Was there clear reporting of the demographics of the participants in the study?, it. 7= Was there clear reporting of clinical information of the participants?, it. 8= Were the outcomes or follow-up results of cases clearly reported?, it. 9= Was there clear reporting of the presenting sites'/clinics' demographic information?, and it. 10= Was statistical analysis appropriate?. In order to provide clear identification of the results presented in the table, the final output was adjusted to correspond with the color scheme used by Cochrane's Risk of Bias 2 tool. Specifically, "Yes" was represented by the color green, "Unclear" by yellow, and "No" by red.

The JBI was considered in the interpretation of the results by applying the Grading of Recommendations Assessment, Development and Evaluation (GRADE) system. Briefly, the overall quality was rated as high and downgraded one level to moderate, low, or very low for each of the following limitations. The reason for downgrading the evidence is summarised in Table 1 Supplementary File 3.

Table. Reasons to downgrade the level of evidence.

Domain	Reason to downgrade the level of evidence
**Imprecision (-1)**	• if the conclusion about the effect magnitude would be altered based on the lower or upper boundary of the confidence interval. For example, if the mean effect was small, but the 95% confidence interval crossed the threshold for a trivial effect (i.e., $g < 0.2$), the precision was insufficient to support a strong recommendation the confidence interval does not exclude the possibility for the effect to be trivial. Similarly, if the confidence interval crossed the threshold for a large effect, while the mean effect was moderate, the conclusion was considered imprecise and as such, the level of evidence was also downgraded one level.
**Inconsistency (-1)**	• if large statistical heterogeneity was observed (e.g., $I^2 > 50\%$) when standardized mean differences were calculated.
**Risk of bias (-1)**	• if most studies (> 65%) rated as being at unclear risk of bias<br>• outcome includes studies that have been rated as being at high risk of bias in two or more categories
