# Supplementary material for: Radiological and functional outcomes of Reverdin Isham osteotomy in moderate Hallux Valgus: a systematic review and meta-analysis
Source: Sci Rep. 2024 Jun 26;14:14781. doi: 10.1038/s41598-024-65440-3 (PMC11208448; doi:10.1038/s41598-024-65440-3)
Supplement: Supplementary file 4 — Supplementary Information 4. [file 41598_2024_65440_MOESM4_ESM.pdf]

Supplementary File 4. Effect sizes calculations and meta-analysis procedures**.**

To calculate effect size and variance of AOFAS scores, IMA, DMAA and HV_{angle} the following equations were used:

$$(A)SMD = c(df_{Exp}) \cdot \left[\frac{(\bar{X}_{pre,Exp}) - (\bar{X}_{post,Exp})}{\bar{S}_{pre}}\right]$$

Where "df_{exp}" refers to degree of freedom of experimental group and "$\bar{S}_{pre}$" is the standard deviation of the experimental group in the pre-test. And "c" is the correction factor, which was obtained by:

$$c(df_{Exp}) = 1 - \left[\frac{3}{4\,(n_{exp} - 1) - 1}\right]$$

The variance of the SMD was computed as:

$$S_{SMD}^2 = [c(df_{Exp})]^2 . \left[\frac{2 * (1 - r)}{n}\right] \cdot \left[\frac{n - 1}{n - 3}\right] \cdot \left(1 + \frac{n * SMD_{exp}^2}{2 * (1 - r)}\right) - SMD_{exp}^2$$

Where "r" is the average correlation coefficient between the pre and post measurements. The correlation coefficient between pre-post measurements was computed from the standard deviation of change score (SD_{diff}), with SD_{diff} being determined as detailed by en Higgins et al. (1) . If the reported outcomes could not be used to calculate SD_{diff}, the corresponding author of each paper was contacted to provide the data. The SMD was considered trivial (< 0.20), small (0.20 – 0.59), moderate (0.60 – 1.19), large (1.20 – 1.99), and very large (> 2.00).

Variance estimations between studies were calculated using a random effects model (i.e., Hartung-Knapp/Sidik-Jakman adjustment [HKSJ]) with a 95% confident interval ($CI_{95\%}$). The consistency of the effects found was assessed using the heterogeneity (I^2) and Tau-square tests (τ^2) tests, with I^2 being considered small (<25%), moderate (25 – 49%) and high (>50%). In addition, τ^2 and prediction interval (PI) were included, because τ^2 cannot readily point to the clinical implications of the unobserved heterogeneity (2). The prediction interval allows a better clinical evaluation of the results obtained because it represents the range in which the effect size of a future study conducted on the topic will most likely be (i.e., probability of true-positive effect). Prediction intervals and the probability of the true-positive effects calculations were performed in accordance with IntHout, Ioannidis and Goeman (2). All statistical analyses were performed using statistical software (R version 4.1.9, metaphor and meta-analysis package, general meta-analysis package).

Bibliografía

1. Higgins JPT, López-López JA, Becker BJ, Davies SR, Dawson S, Grimshaw JM, et al. Synthesising quantitative evidence in systematic reviews of complex health interventions. BMJ Glob Heal [Internet]. 2019 [cited 2023 Aug 4];4:858.

2. Inthout J, Ioannidis JPA, Rovers MM, Goeman JJ. Plea for routinely presenting prediction intervals in meta-analysis.
