# Supplementary material for: Radiological and functional outcomes of Reverdin Isham osteotomy in moderate Hallux Valgus: a systematic review and meta-analysis
Source: Sci Rep. 2024 Jun 26;14:14781. doi: 10.1038/s41598-024-65440-3 (PMC11208448; doi:10.1038/s41598-024-65440-3)
Supplement: Supplementary file 5 — Supplementary Information 5. [file 41598_2024_65440_MOESM5_ESM.pdf]

Supplementary File 5. Eliminates studies with reasons

Reason 1. Some data not available

1. Burg, A., & Palmanovich, E. (2020). Correction of Severe Hallux Valgus with Metatarsal Adductus Applying the Concepts of Minimally Invasive Surgery. Foot and Ankle Clinics, 25(2), 337–343.
2. Burg, A., Tal Frenkel, R., Nyska, M., Ohana, N., Segal, D., & Palmanovich, E. (2022). Treating Hallux Valgus Associated Metatarsus Adductus by Minimally Invasive Surgery: A Simple Solution for a Difficult Problem. The Journal of Foot and Ankle Surgery : Official Publication of the American College of Foot and Ankle Surgeons, 61(5), 1091–1097.
3. Isham, S. A. (1991). The Reverdin-Isham procedure for the correction of hallux abducto valgus. A distal metatarsal osteotomy procedure. Clinics in Podiatric Medicine and Surgery, 8(1), 81–94.
4. Luria, T., Dudkiewicz, I., Burg, A., Heller, S., Salai, M., & Tytiun, Y. (2010). The early results of minimally invasive technique for hallux valgus repair. Foot (Edinburgh, Scotland), 20(4), 118–120.

Reason 2. Other surgical techniques

1. Aiyer, A., Massel, D. H., Siddiqui, N., & Acevedo, J. I. (2021). Biomechanical Comparison of 2 Common Techniques of Minimally Invasive Hallux Valgus Correction. Foot & Ankle International, 42(3), 373–380.
2. Botezatu, I., Marinescu, R., & Laptoiu, D. (2015). Minimally invasive-percutaneous surgery—Recent developments of the foot surgery techniques. Journal of Medicine and Life, 8 Spec Issue, 87–93.
3. Brogan, K., Voller, T., Gee, C., Borbely, T., & Palmer, S. (2014). Third-generation minimally invasive correction of hallux valgus: Technique and early outcomes. International Orthopaedics, 38(10), 2115–2121.
4. Choi, J. Y., Ahn, H. C., Kim, S. H., Lee, S. Y., & Suh, J. S. (2019). Minimally invasive surgery for young female patients with mild-to-moderate juvenile hallux valgus deformity. Foot and Ankle Surgery : Official Journal of the European Society of Foot and Ankle Surgeons, 25(3), 316–322.

5. De Lavigne, C., Rasmont, Q., & Hoang, B. (2011). Percutaneous double metatarsal osteotomy for correction of severe hallux valgus deformity. Acta Orthopaedica Belgica, 77(4), 516–521.
6. Del Castillo, J., Russi, M., Filomeno, P., Kenny-Pujadas, J. E., Cabrera-Frola, J. A., Fischer, A., Bongiovanni, J. C., & Amaya, J. (2021). [Experimental cadaveric study assesing protection and osteotomy guide system (BARU) in hallux valgus surgery using Reverdin-Isham technic]. Acta Ortopedica Mexicana, 35(2), 132–136.
7. Di Giorgio, L., Sodano, L., Touloupakis, G., De Meo, D., & Marcellini, L. (2016). Reverdin-Isham osteotomy versus Endolog system for correction of moderate hallux valgus deformity: A Randomized Controlled Trial. La Clinica Terapeutica, 167(6), e150–e154.
8. Díaz Fernández, R. (2015). [Treatment of moderate and severe hallux valgus by performing percutaneous double osteotomy of the first metatarsal bone]. Revista Espanola de Cirugia Ortopedica y Traumatologia, 59(1), 52–58.
9. Frigg, A., Zaugg, S., Maquieira, G., & Pellegrino, A. (2019). Stiffness and Range of Motion After Minimally Invasive Chevron-Akin and Open Scarf-Akin Procedures. Foot & Ankle International, 40(5), 515–525.
10. Hernandez, J. L. y, Golano, P., Roshan-Zamir, S., Darcel, V., Chauveaux, D., & Laffenetre, O. (2016). Treatment of moderate hallux valgus by percutaneous, extra-articular reverse-L Chevron (PERC) osteotomy. 98B(3), 365–373.
11. Holme, T. J., Sivaloganathan, S. S., Patel, B., & Kunasingam, K. (2020). Third-Generation Minimally Invasive Chevron Akin Osteotomy for Hallux Valgus. Foot & Ankle International, 41(1), 50–56.
12. Kadakia, A. R., Smerek, J. P., & Myerson, M. S. (2007). Radiographic results after percutaneous distal metatarsal osteotomy for correction of hallux valgus deformity. Foot & Ankle International, 28(3), 355–360.
13. Lewis, T. L., Ray, R., & Gordon, D. J. (2022). Minimally invasive surgery for severe hallux valgus in 106 feet. Foot and Ankle Surgery : Official Journal of the European Society of Foot and Ankle Surgeons, 28(4), 503–509.
14. Lim, W. S. R., Rikhraj, I. S., & Koo, K. O. T. (2021). Simultaneous bilateral hallux valgus surgery: Percutaneous or conventional? Early results of a matched study from a tertiary institution. Foot and Ankle Surgery : Official Journal of the European Society of Foot and Ankle Surgeons, 27(4), 377–380.

15. Neufeld, S. K., Dean, D., & Hussaini, S. (2021). Outcomes and Surgical Strategies of Minimally Invasive Chevron/Akin Procedures. Foot & Ankle International, 42(6), 676–688.
16. Stamatis, E. D., Huber, M. H., & Myerson, M. S. (2004). Transarticular distal soft-tissue release with an arthroscopic blade for hallux valgus correction. Foot & Ankle International, 25, 13–18.

Reason 3 They use osteosynthesis material

1. Bauer, T. (2014). Percutaneous forefoot surgery. Orthopaedics & Traumatology, Surgery & Research : OTSR, 100, S191-204.
2. Bauer, T., de Lavigne, C., Biau, D., De Prado, M., Isham, S., & Laffenétre, O. (2009). Percutaneous hallux valgus surgery: A prospective multicenter study of 189 cases. The Orthopedic Clinics of North America, 40(4), 505–514, ix.
3. Crespo Romero, E., Arcas Ordoño, A., Peñuela Candel, R., Gómez Gómez, S., Arias Arias, A., Gálvez González, J., & Crespo Romero, R. (2017). Percutaneous Hallux Valgus Surgery Without Distal Metatarsal Articular Angle Correction. Foot & Ankle Specialist, 10(6), 502–508.
4. Crespo Romero, E., Peñuela Candel, R., Gómez Gómez, S., Arias Arias, A., Arcas Ordoño, A., Gálvez González, J., & Crespo Romero, R. (2017). Percutaneous forefoot surgery for treatment of hallux valgus deformity: An intermediate prospective study. Musculoskeletal Surgery, 101(2), 167–172.
5. Enan, A., Abo-Hegy, M., & Seif, H. (2010). Early results of distal metatarsal osteotomy through minimally invasive approach for mild-to-moderate hallux valgus. Acta Orthopaedica Belgica, 76(4), 526–535.
6. Kaufmann, G., Handle, M., Liebensteiner, M., Braito, M., & Dammerer, D. (2018). Percutaneous minimally invasive Akin osteotomy in hallux valgus interphalangeus: A case series. International Orthopaedics, 42(1), 117–124.
7. Liszka, H., & Gądek, A. (2020). Percutaneous Transosseous Suture Fixation of the Akin Osteotomy and Minimally Invasive Chevron for Correction of Hallux Valgus. Foot & Ankle International, 41(9), 1079–1091.
