# Supplementary material for: Radiological and functional outcomes of Reverdin Isham osteotomy in moderate Hallux Valgus: a systematic review and meta-analysis
Source: Sci Rep. 2024 Jun 26;14:14781. doi: 10.1038/s41598-024-65440-3 (PMC11208448; doi:10.1038/s41598-024-65440-3)
Supplement: Supplementary file 6 — Supplementary Information 6. [file 41598_2024_65440_MOESM6_ESM.pdf]

Table 1. Supplementary File 6. Characteristics of studies investigated the effect of Reverdin Isham Osteotomy for Moderate Hallux Abducts Valgus patients.

										**Reverdin and Akin**			**Complications**		**Conclusions**
**Study**	**Sample Size**	**Age**	**Sex**	**Body mass (kg)**	**Study period**	**Country**	**Study Design**	**SIGN**	**Consent**	**Intervention type**	**Severity**	**Incision Length (mm)**	**Minor**	**Major**	**Main findings**
Biz et al.	80	51	5 M<br>75 F	NR	May 2010 to May 2012	Padova, Italy	Case study	IV	YES	Exostectomy + R-I+ Akin + ADD tenotomy + lateral capsulotomy	Mild to Severe	3-5 mm (R-I)<br>2-3 mm ADD 3-5 mm Akin	Recurrence (5), severe stifness (1)	Slighly loss normal range of MTP joint (16), delayed wound healing (3)	Reverdin isham and akin with the exostectomy and the release of soft parts are sure, cash and reliable for correction of HV<br>symptomatic of mild to moderate.
Restuccia et al.	124	62.5	NR	NR	May 2011 to December 2015	Italy	Case study	IV	YES	Exostectomy + R-I+ Akin + ADD tenotomy + lateral capsulotomy	Mild to Severe	3 mm (R-I)<br>3 mm ADD	No complication	Incomplete correction of deformity (11), transfer metatarsalgia (12), overcorrection HV (2)	Forefoot surgery percutaneous without osteosynthesis provides a quick one recovery functional with pain postoperative mild to moderate.
Ribeiro et al.	79	56.4	63 M<br>6 F	NR	June 2010 to July 2019	Brazil	Case study	IV	YES	Exostectomy + R-I+ Akin + ADD tenotomy + lateral capsulotomy	Mild Moderate	5-8 mm (R-I)<br>2 mm ADD<br>3-5 mm Akin	Recurrence (1), third metatarsal stress fracture (1)	Hallux hypoesthesia (2), transfer metatarsalgia (1), type 1 complex regional pain syndrome (3)	The modification<br>of the osteotomy M1 in technique RI evidenced results significant in as for his stability.
Severyns et al.	57	51.5	5 M<br>43 F	NR	May 2003 to November 2011	France	Case study	IV	NR	Exostectomy + R-I+ Akin + ADD tenotomy + lateral capsulotomy	Mild Moderate	3 mm (R-I)	Deep vein thrombosis (1)	Transfer metatarsalgia (4), cutaneous delayed heal-ing (5), recurrences (2), hallux hypoesthesia (3)	Reverdin<br>isham osteotomy is safe and effective for symptomatic hallux abductus valgus moderate.
Rodriguez -Reyes et al.	20	46.9	1 M<br>10 F	67.4	NR	Mexico	Case study	IV	NR	Exostectomy + R-I+ Akin + ADD tenotomy + lateral capsulotomy	Mild Moderate	NR	NR	NR	Reverdin- isham for correction of hallux valgus It allows get results satisfactory in lineup structural bone and aesthetic of the foot.
Bauer et al. 2009	179	55	4 M<br>164 F	NR	September 2005 to February 2006	France	Case study	IV	NR	Exostectomy + R-I+ Akin + ADD tenotomy + lateral capsulotomy	Mild Moderate	3-5 mm (R-I)<br>3mm Akin	Deep vein thrombosis (3), first MTP joint stiffness (2)	Type 2 complex regional pain syndrome (5)	The results clinical obtained with this technique percutaneous for the correction of moderate HAV are comparable to those obtained other techniques percutaneous.
Bauer et al.<br>2010	104	NR	NR	NR	September 2004 to June 2006	France	Case study	IV	NR	Exostectomy + R-I+ Akin + ADD tenotomy + lateral capsulotomy	Mild Moderate	3-5 mm (R-I)<br>3mm Akin	Fracture (9), first MTP joint stiffness (2), recurrence (3)	DMMA overcorrection (6), complex regional pain syndrome (2), tranfer metatarsalgia (2)	Reverdin isham provides results clinical satisfactory. However, requires of a curve of learning elderly.

Note: NR= , R-I= Reverdin Isham, M= male, F= female, ADD= adductor,
